# Adversity coping capability and its associations with mental health and family wellbeing amid the COVID-19 pandemic in Hong Kong

**DOI:** 10.1186/s12888-022-04198-2

**Published:** 2022-08-12

**Authors:** Wei Jie Gong, Shirley Man Man Sit, Agnes Yuen Kwan Lai, Nancy Xiaonan Yu, Man Ping Wang, Sai Yin Ho, Tai Hing Lam

**Affiliations:** 1 Department of General Practice, Health Science Center, Shenzhen University, Shenzhen, China; 2 School of Public Health, The University of Hong Kong, Hong Kong, China; 3 School of Nursing, The University of Hong Kong, Hong Kong, China; 4 Department of Social and Behavioral Sciences, City University of Hong Kong, Hong Kong, China

**Keywords:** Adversity coping capability, Resilience, COVID-19, Mental health, Family wellbeing

## Abstract

**Background:**

Adversity coping capability (ACC) is important amid the COVID-19 pandemic. We examined the associations of ACC as measured by our one-item ACC scale (ACC-1) with mental health, family well-being and validity of ACC-1 in Hong Kong.

**Methods:**

A cross-sectional survey was conducted on Hong Kong Chinese adults aged ≥ 18 years by landline, mobile phone, and online survey from February to March 2021, when the fourth wave of COVID-19 was under control. ACC-1 consisted of the question: “How do you rate your capability to cope with adversities?” with higher scores (0–10) indicating stronger ACC. The associations of ACC with socioeconomic characteristics, resilience, mental health, and family wellbeing were examined by linear regression coefficients (*β*s). Data were weighted by sex, age, and education of the general population.

**Results:**

Of 7441 respondents, after weighing, 52.2% were female and 79.1% were aged 18 to 64 years. ACC-1 showed good construct validity, with higher ACC being associated with higher levels of resilience (adjusted *β* = 0.29), personal happiness (0.55), family happiness (0.42), family wellbeing (0.41), and family communication quality (0.41), and lower levels of depressive symptoms (-0.30), anxiety (-0.30), loneliness (-0.15); incremental validity with additional contributions of ACC to mental health and family wellbeing; and known-group validity with older age and favorable socioeconomic characteristics showing higher ACC (all *P* < 0.02). Females (mean ± standard deviation: 6.04 ± 1.82 vs 6.15 ± 1.96 [male]) and unemployed respondents (5.30 ± 1.99 vs 6.11 ± 2.03 [in paid employment]) had lower ACC (all *P* ≤ 0.02).

**Conclusions:**

We have first shown that stronger ACC was associated with better mental health and family wellbeing, and the results support ACC-1 as a simple and valid measure of ACC.

## Background

The coronavirus (COVID-19) pandemic has posed significant social, economic, and health challenges globally. Disruptions to daily routines, physical isolation, and financial insecurity have caused an increase in global mental health burdens [1]. The adversity caused by COVID-19 may vary among individuals, in which those with well-functioning families, more socioeconomic resources, and strong coping capability are less likely to be affected [2]. Hong Kong, one of the most developed cities in China, is also among the cities with the highest socioeconomic disparities [3]. Almost 100% voluntary mask-wearing in public shortly after the first reported case, stringent social distancing regulations with no lock-down, and other elimination measures facilitated the successful control of the first 4 waves of outbreaks in Hong Kong [3, 4]. However, mental health crises were still reported under in the city, including increased stress levels, higher prevalence of anxiety, and doubled prevalence of depression and unhappiness during the COVID-19 outbreak [5, 6].

When individuals face disruptive challenges and endure difficult transition, adversity coping is the changeable process of making cognitive and behavioral efforts to manage the demands of stressful events considered taxing or exceeding one’s resources [3, 7]. According to Lazarus and Folkman’s stress and coping theory, coping refers to the emotions and thoughts that individuals use to manage distress (emotion-focused coping), the actions to deal with the problem causing the distress (problem-focused coping), and the meaning-making to sustain positive wellbeing (meaning-focused coping) [7, 8]. Individuals use an array of these coping strategies in real-life situations [9]. Adversity coping capability (ACC) is an individual’ perceived capability to cope with an adversity or stressful event. It is influenced by coping resources including psychological, social, environmental, and spiritual resources, as well as the nature of the adverse situation [9], and has significant influences on stress-related physical and mental health outcomes [7, 10].

The Chinese terms of ACC, ‘抗逆力’ or ‘抗逆能力’, were frequently used as the translations of resilience in the Hong Kong media and even in interventions and social policies by organizations. Our search on 13 February 2022 using the two terms in Google (9,890,000 results), Yahoo (528,000 results), Microsoft Bing (882,000 results), and Baidu (29,140,000 results) yielded 40,440,000 results. Resilience is a more complex concept that extends beyond adversity coping, involving positive adapting, and bouncing back or recovering from adversity [11–13]. The more literally accurate Chinese translations of resilience, ‘反彈力’ and ‘復原力’, were much less used in the media and by organizations. Our search of these terms on 13 February 2022 in Google (669,000 results), Yahoo (791,000 results), Microsoft Bing (459,000 results), and Baidu (24,632,000 results) yielded 1,919,000 results. Another Chinese translation of resilience, ‘心理彈性’, is commonly used in academic settings but difficult for laypersons to understand.

The World Health Organization has warned there would be no return to ‘the old normal’ amid COVID-19 [14]. As ACC is important for positive coping with adversities and challenges brought by the pandemic and has become a common concern, with the term being widely used in Hong Kong, it is necessary to assess coping using a valid scale amid the pandemic. Previous frequently used scales were developed to comprehensively measure subscales of coping, such as Carver’s 53-item COPE scale [15], whereas long questionnaires would cause high attrition rates and more refusals to answer [16]. Therefore, we developed a simple and direct one-item ACC scale (ACC-1) with an 11-point Likert scale ranging from 0 to 10. Based on a large-scale population-based survey amid the pandemic, this study aimed to assess ACC-1 and its socioeconomic disparities in Hong Kong Chinese adults amid the pandemic, to analyze the associations of ACC-1 with resilience, mental health, and family wellbeing, and to assess the validity of ACC-1 by examining its psychometric properties. The concise ACC-1 scale could minimize the response burden in large surveys. The results would show the disparities in individuals’ coping capability amid the pandemic and present the validity of ACC-1.

## Methods

### Sample and procedures

This is a cross-sectional study. Under the Jockey Club SMART Family-Link project, we conducted the population-based Family amid COVID-19 Survey 2 (FamCov-2) from 22 February to 23 March 2021 on Hong Kong residents aged ≥ 18 years who were able to read or communicate using Cantonese, aiming to recruit as many respondents as possible within 4 weeks when the fourth wave of the COVID-19 pandemic was under control in Hong Kong. Hong Kong Public Opinion Research Institute (HKPORI), a well-known local survey agency, was commissioned to conduct the survey using landline telephone, mobile telephone, and online methods.

The survey design and methods have been published [17]. All phone numbers were randomly generated using known prefixes assigned to telecommunication service providers under the Numbering Plan of the Office of the Communication Authority. The invalid numbers were eliminated to produce a final telephone sample. For the landline telephone survey, a second-level sampling was adopted. Only one eligible respondent was selected among all those present in a household using the ‘next birthday rule’ (i.e., select the qualified family member whose next birthday is nearest to the interview date). No second-level sampling was used in the mobile sample. Each telephone interview took around 10 min. Of the 1604 and 816 qualified respondents who answered the landline and mobile telephone surveies, respectively, 1022 (63.7%) and 500 (61.2%) respondents completed the whole survey, respectively. The online survey was self-administered and sent to HKPORI’s probability and non-probability online panel members by email invitations, with an access link to the survey website [17]. Among the 4,311 and 44,514 probability and non-probability panel members who opened the invitation emails, 641 (14.2%) and 5372 (12.1%) respondents completed the whole survey, respectively.

### Measures

#### ACC

*ACC* was measured using the item ‘How do you rate your capability to cope with adversities?’ on an 11-point Likert scale from 0 to 10 (0 = no capability, 5 = half-half, 10 = very high capability).

#### Resilience

*Resilience* was measured using the 2-item abbreviated version of the Connor-Davidson Resilience Scale (CD-RISC2), and only answered by respondents in the landline survey. It consists of two items, ‘Able to adapt to change’ (CD-RISC2-a, for adaptation ‘適應’) and ‘Tend to bounce back after illness or hardship’ (CD-RISC2-r, for recovery ‘恢復’), on a 5-point Likert scale of ‘never’, ‘seldom’, ‘sometimes’, ‘often’, and ‘almost all the time’ [18], with good reliability and validity in Hong Kong [19]. The total score ranged from 0 to 10, with higher scores indicating higher levels of resilience.

#### Mental health

*Depressive symptoms* were measured using the 2-item Patient Health Questionnaire (PHQ-2) [20], and a*nxiety symptoms* were measured using the 2-item General Anxiety Disorder (GAD-2) [20]. The scores for both symptoms ranged from 0 to 6, with higher scores indicating more symptoms. *Loneliness* was measured using a single item, ‘Over the past 7 days, how often do you feel lonely?’ with responses of 0, 1–2, 3–4, and 5–7 days [21]. *Personal happiness* was measured using a single item, ‘How happy do you think you are?’, on an 11-point scale from 0 (very unhappy) to 10 (very happy), with proven reliability and validity [22].

#### Family wellbeing

In this survey, family was defined as ‘family members who are related through biological, marital, cohabitation, and/or emotional bonding’, which was explained to the respondents before asking questions related to family. *Family happiness* was measured by our single-item Self-reported Family Happiness Scale of ‘How happy do you think your family is?’, with reported reliability and validity in Hong Kong Chinese [23]. *Family wellbeing* was calculated as the mean score of family 3H (happiness, health, and harmony) using the questions ‘How happy/healthy/harmonious do you think your family is?’. *Family communication quality* was measured using the single item ‘How do you find the quality of communication between you and your family members?’. All these measures were rated on an 11-point scale of 0 to 10 and have been used in our previous studies, with higher scores indicating higher overall family happiness, wellbeing or communication quality [24, 25].

Information of sociodemographic characteristics was also collected, including sex, age, education, employment status, monthly household income, housing type (rented/owned), and the number of cohabitants.

### Statistical analysis

All data were weighted by the sex, age, and education distribution of the 2019 Hong Kong general adult population from the Census and Statistics Department [26, 27], using the random iterative methods.

Linear regressions were used to calculate crude and adjusted regression coefficients (*β*s) and their 95% confidence intervals (CIs) for the associations of ACC with socioeconomic characteristics, with the latter being mutually adjusted, and for the associations of ACC with resilience, mental health, and family wellbeing, with adjustments of socioeconomic characteristics. As a minimum of two subjects per variable is required for adequate estimation of regression coefficients [28], considering the number of categories in each independent variable, including 2 categories for sex, education, and housing type, 3 categories for monthly household income and number of cohabitants, 4 categories for age, and 5 categories for employment status, the minimum sample size required in the planned linear regressions was 2*(2*3 + 3*2 + 4 + 5) = 42.

Standardized Cronbach’s α was calculated to assess the internal reliability of CD-RISC2, GAD-2, and PHQ-2, as it is more appropriate for two-item scales [29].

#### Validity of ACC-1

To examine the construct validity (convergent and discriminant validity), we calculated the zero-order correlation of ACC-1 with CD-RISC2, mental health and family wellbeing by sex, age, and education using Pearson correlation coefficients (Pearson *r*), including resilience, depressive symptoms, anxiety symptoms, loneliness, personal happiness, family happiness, family wellbeing (happiness, health, and harmony) and family communication quality [19, 30–33]. We hypothesized that ACC would be negatively correlated with depressive symptoms, anxiety symptoms, and loneliness [30–32], and positively correlated with resilience [33], personal happiness, family happiness, family wellbeing, and family communication quality [19, 34]. Pearson *r* of 0.10, 0.30, and 0.50 were considered as small, medium, and strong associations respectively [35].

To examine the divergent validity that ACC-1 measures a different construct to resilience measured using the CD-RISC2, we calculated the Pearson correlation coefficients (r) of the ACC-1, CD-RISC2, CD-RISC2-a, and CD-RISC2-r with measures of mental health and family wellbeing. We used Pearson and Filon's *z* test to compare Pearson *r* [36].

To examine the incremental validity, after accounting for sociodemographic variables including sex, age, education, employment status, monthly household income, and housing type (Step 1), a set of hierarchical regression analyses were conducted to estimate the additional contribution of ACC-1 (Step 3) over the CD-RISC2 (Step 2) in the estimations of anxiety and depression symptoms, loneliness, personal happiness, and family happiness, wellbeing, and communication quality. We also repeated the analysis by separately including CD-RISC2-a and CD-RISC2-r in Step 2 (i.e. Step 2’) and ACC-1 in Step 3 (i.e. Step 3’).

To examine the known-group validity, ACC-1 mean scores were compared across different socioeconomic characteristics using univariate regression analysis. Based on previous evidence [37, 38], we hypothesized that those with favorable socioeconomic status, including higher education, in paid employment, higher household income, and owned housing, would have higher ACC. All analyses were conducted using STATA 15.0 (StataCorp LP, College Station, TC, USA). A two-tailed *P* < 0.05 indicated statistical significance.

## Results

Totally 7535 respondents were enrolled in the landline telephone, mobile telephone, and online surveys. Of them, ACC-1 was answered by 7441 (98.8%) respondents. The standardized Cronbach’s α for CD-RISC2, GAD-2, and PHQ-2 was 0.65, 0.82, and 0.79, respectively. Table 1 shows that 52.2% of ACC-1 respondents were female, 79.1% were aged 18–64 years, 35.3% were tertiary educated, 55.5% had in-paid employment, and 49.6% had a monthly household income of HK$10,000–39,999 (US$1 = HK$7.8). After mutual adjustment, female and unemployed respondents had lower scores of ACC-1 (all *P* ≤ 0.02). Respondents aged ≥ 25 years, had tertiary education, had higher monthly household income (≥ HK$ 10,000), or were retired, had higher scores of ACC-1 (all *P* < 0.003).

**Table 1:** Sociodemographic characteristics of respondents and their associations with ACC ^a^

Characteristics	Unweighted n (%)	Weighted^b^ n (%)	Crude *β* (95% CI)	Adjusted *β* (95% CI)^c^
Sex				
Male	3614 (48.6)	3554 (47.8)	0	0
Female	3827 (51.4)	3879 (52.2)	-0.18 (-0.26, -0.09)***	-0.11 (-0.20, -0.02)*
Age, years				
18–24	588 (7.9)	658 (8.9)	0	0
25–44	3016 (40.6)	2433 (32.8)	0.29 (0.13, 0.46)***	0.26 (0.02, 0.50)*
45–64	2864 (38.6)	2853 (38.5)	0.64 (0.47, 0.80)***	0.68 (0.43, 0.93)***
≥ 65	958 (12.9)	1475 (19.9)	0.64 (0.45, 0.83)***	0.78 (0.48, 1.09)***
Education				
Secondary or below	2067 (28.0)	4775 (64.7)	0	0
Tertiary	5324 (72.0)	2610 (35.3)	0.23 (0.14, 0.33)***	0.26 (0.14, 0.37)***
Employment status				
In paid employment	4822 (65.8)	4071 (55.5)	0	0
Unemployed	411 (5.6)	467 (6.4)	-0.63 (-0.82, -0.45)***	-0.34 (-0.55, -0.14)**
Retired	1229 (16.8)	1716 (23.4)	0.22 (0.10, 0.33)***	0.23 (0.06, 0.40)**
Housekeeper	492 (6.7)	652 (8.9)	-0.10 (-0.28, 0.07)	0.07 (-0.14, 0.27)
Full-time students	379 (5.2)	425 (5.8)	-0.49 (-0.68, -0.29)***	0.01 (-0.28, 0.30)
Monthly household income, HKD				
< 10,000	741 (11.6)	957 (14.6)	0	0
10,000–39,999	2442 (38.1)	3240 (49.6)	0.25 (0.10, 0.40)**	0.47 (0.31, 0.64)***
≥ 40,000	3221 (50.3)	2339 (35.8)	0.74 (0.59, 0.89)***	0.85 (0.68, 1.03)***
Housing type				
Rented	2864 (38.7)	3077 (41.6)	0	0
Owned	4530 (61.3)	4315 (58.4)	0.26 (0.17, 0.35)***	0.06 (-0.03, 0.16)
Number of cohabitants				
0	639 (8.6)	588 (7.9)	0	0
1–3	5733 (77.1)	5667 (76.3)	0.07 (-0.08, 0.22)	-0.02 (-0.20, 0.16)
≥ 4	1062 (14.3)	1172 (15.8)	0.17 (-0.01, 0.35)	0.13 (-0.08, 0.34)

*ACC* Adversity coping capability, range 0–10, *CI* Confidence interval. US$1 = HK$7.8

^a^*n* = 7441, respondents with missing data were excluded. Percentages may not total 1 after rounding

^b^Weighted by the sex, age, and education distribution of 2019 Hong Kong general population

^c^Mutually adjusted for each other

^*^ *P* < 0.05, ** *P* < 0.01, *** *P* < 0.001

Table 2 shows the associations of ACC with resilience and wellbeing. After adjusting for socioeconomic characteristics, respondents with higher ACC-1 scores showed higher levels of resilience, personal happiness, family happiness, family wellbeing, and family communication quality (Adjusted *β*: 0.29 to 0.55), as well as lower levels of anxiety symptoms, depression symptoms, and loneliness (Adjusted *β*: -0.30 to -0.15) (all *P* < 0.001).

**Table 2:** Associations of ACC with resilience, mental health, and family wellbeing^a^

	Crude *β* (95% CI)	Adjusted *β* (95%CI)^c^
Resilience^b^	0.33 (0.28, 0.38)***	0.29 (0.23, 0.35)***
Depressive symptoms	-0.33 (-0.34, -0.31)***	-0.30 (-0.32, -0.28)***
Anxiety symptoms	-0.32 (-0.34, -0.30)***	-0.30 (-0.32, -0.28)***
Loneliness	-0.17 (-0.18, -0.16)***	-0.15 (-0.17, -0.14)***
Personal happiness	0.56 (0.54, 0.58)***	0.55 (0.52, 0.57)***
Family happiness	0.44 (0.42, 0.46)***	0.42 (0.39, 0.44)***
Family wellbeing	0.43 (0.41, 0.45)***	0.41 (0.38, 0.43)***
Family communication quality	0.43 (0.41, 0.46)***	0.41 (0.38, 0.43)***

*ACC* Adversity coping capability, range 0–10, *CI* Confidence interval, US$1 = HK$7.8

^a^*n* = 7441, missing data were excluded

^b^*n* = 937 for resilience, only answered in the landline survey

^c^Adjusted for all sociodemographic characteristics, including sex, age, education, employment status, monthly household income, housing type, and number of cohabitants

^*^P < 0.05, **P < 0.01, ***P < 0.001

For the construct validity of ACC-1, Table 3 shows that ACC-1 was correlated with all other measures in the hypothesized directions. Specifically, ACC-1 was negatively correlated with anxiety symptoms, depression symptoms, and personal loneliness (*r* = -0.40 to -0.33), and positively correlated with personal happiness, CD-RISC2, CD-RISC2-a, CD-RISC2-b, family happiness, family wellbeing, and family communication quality (*r* = 0.28 to 0.50) (all *P* < 0.001). The directions of these correlations were consistent across sex, age, and education.

**Table 3:** The correlations of ACC with resilience, mental health, and family wellbeing by socioeconomic characteristics^a^

	Overall	Sex		Age, years				Education	
		Male	Female	18–24	25–44	45–64	65 +	Secondary or below	Tertiary
Resilience^b^	0.37	0.31	0.42	0.25^c^	0.48	0.39	0.36	0.34	0.46
Depressive symptoms	-0.40	-0.42	-0.38	-0.38	-0.38	-0.42	-0.32	-0.38	-0.42
Anxiety symptoms	-0.38	-0.39	-0.37	-0.36	-0.35	-0.41	-0.32	-0.36	-0.41
Loneliness	-0.33	-0.38	-0.28	-0.26	-0.32	-0.34	-0.30	-0.32	-0.34
Personal happiness	0.50	0.51	0.51	0.39	0.49	0.54	0.50	0.50	0.52
Family happiness	0.40	0.43	0.38	0.27	0.37	0.44	0.37	0.41	0.40
Family wellbeing	0.41	0.44	0.39	0.29	0.38	0.46	0.37	0.43	0.41
Family communication quality	0.37	0.39	0.36	0.33	0.34	0.42	0.33	0.40	0.38

*ACC* Measured using the one-item adversity coping capability scale

^a^*n* = 7441, missing data were excluded

^b^*n* = 937 for resilience, only answered in the landline survey

All were Pearson correlation coefficients (*r*), and all the corresponding *P* < 0.001 except ^c ^*P* < 0.05

For divergent validity, Table 4 shows that, except for depressive symptoms and loneliness, the correlations of all other measures with ACC-1 were significantly stronger than with CD-RISC2, CD-RISC2-a, and CD-RISC2-b (all *P* ≤ 0.04). The correlation of ACC-1 with CD-RISC2-a (*r* = 0.35) was significantly stronger than with CD-RISC2-r (*r* = 0.28) (*P* = 0.02).

**Table 4:** The correlations of ACC-1 and CD-RISC2 with mental health and family wellbeing (*n* = 937)

	ACC-1	CD-RISC2	*P*^a^	CD-RISC2-a	*P*^a^	CD-RISC2-r	*P*^a^
CD-RISC2	0.37	-	-	-	-	-	-
CD-RISC2-a	0.35	-	-	-	-	0.48	< 0.001
CD-RISC2-r	0.28	-		0.48	< 0.001	-	-
Depressive symptoms	-0.28	-0.29	0.77	-0.25	0.40	-0.24	0.28
Anxiety symptoms	-0.32	-0.25	0.04	-0.24	0.02	-0.20	0.001
Loneliness	-0.29	-0.31	0.56	-0.26	0.40	-0.28	0.42
Personal happiness	0.40	0.31	0.007	0.27	< 0.001	0.26	< 0.001
Family happiness	0.33	0.25	0.02	0.21	0.001	0.23	0.007
Family wellbeing	0.36	0.27	0.008	0.22	0.001	0.24	0.001
Family communication quality	0.32	0.24	0.02	0.20	0.001	0.21	0.003

ACC-1, the one-item adversity coping capability scale

CD-RISC2, the 2-item abbreviated version of the Connor-Davidson Resilience Scale

CD-RISC2-a, ‘Able to adapt to change’, the item for adaptation from CD-RISC2

CD-RISC2-r, ‘Tend to bounce back after illness or hardship’, the item for recovery from CD-RISC2

All *P* values for Pearson correlation coefficients < 0.001

^a^*P* calculated to compare the Pearson r of ACC-1 and CD-RISC2/CD-RISC2-a/CD-RISC2-r with other measures using Pearson and Filon's z test

For incremental validity, Table 5 shows that ACC-1 had additional contributions with all other measures after adjusting for CD-RISC2 and sociodemographic characteristics (all *P* < 0.001). The results were consistent after separately adjusting for CD-RISC2-a and CD-RISC2-r (all *P* < 0.001).

**Table 5:** Adjusted squared multiple regression coefficients for mental health and family wellbeing (*n* = 937)

	Anxiety symptoms	Depressive symptoms	Personal loneliness	Personal happiness	Family happiness	Family wellbeing	Family communication quality
Step 1 R^2^_adj_	0.049	0.044	0.043	0.035	0.050	0.061	0.052
Step 2 R^2^_adj_	0.117	0.124	0.126	0.116	0.105	0.120	0.096
ΔR^2^	0.068	0.080	0.083	0.082	0.056	0.059	0.044
F change	56.15	67.00	69.56	67.85	45.65	49.33	35.88
*P*	< 0.001	< 0.001	< 0.001	< 0.001	< 0.001	< 0.001	< 0.001
Step 3 R^2^_adj_	0.178	0.159	0.159	0.198	0.159	0.185	0.151
ΔR^2^	0.061	0.035	0.033	0.082	0.054	0.065	0.055
F change	54.54	30.27	28.89	74.81	46.83	58.18	47.68
*P*	< 0.001	< 0.001	< 0.001	< 0.001	< 0.001	< 0.001	< 0.001
Step 2’ R^2^_adj_	0.117	0.125	0.127	0.116	0.106	0.120	0.096
ΔR^2^	0.068	0.081	0.085	0.082	0.057	0.059	0.044
F change	28.25	33.62	35.48	33.88	23.23	24.63	17.95
*P*	< 0.001	< 0.001	< 0.001	< 0.001	< 0.001	< 0.001	< 0.001
Step 3’ R^2^_adj_	0.178	0.159	0.162	0.198	0.161	0.185	0.151
ΔR^2^	0.061	0.035	0.034	0.082	0.055	0.065	0.056
F change	54.13	29.97	29.77	74.90	47.78	58.25	48.01
*P*	< 0.001	< 0.001	< 0.001	< 0.001	< 0.001	< 0.001	< 0.001

ACC-1, the one-item adversity coping capability scale

CD-RISC2, the 2-item abbreviated version of the Connor-Davidson Resilience Scale

CD-RISC2-a, ‘Able to adapt to change’, the item for adaptation from CD-RISC2

CD-RISC2-r, ‘Tend to bounce back after illness or hardship’, the item for recovery from CD-RISC2

Step 1, only sociodemographic characteristics including sex, age, education, employment status, monthly household income, housing type, and number of cohabitants (excluding self). Step 2, adding CD-RISC2. Step 3, adding ACC-1

Step 2’, adding CD-RISC2-a and CD-RISC2-r. Step 3’, adding ACC-1

For the known-group validity, Table 6 shows that the mean ACC-1 score in the survey sample was 6.09 ± 1.89. Respondents aged ≥ 45 years, being retired, or with favorable socioeconomic characteristics, including tertiary education, monthly household income ≥ HK$10,000, and owned housing, showed higher ACC-1 scores (all *P* < 0.001). Unemployed respondents and full-time students showed lower ACC-1 scores (both *P* < 0.001). The difference of ACC-1 scores by sex showed no statistical significance (*P* = 0.08).

**Table 6:** Scores of ACC-1 and CD-RISC2 by sociodemographic characteristics, Mean ± SD (95% CI)

Sociodemographic characteristics	ACC-1 (*n* = 7441)	CD-RISC2 (*n* = 937)
Overall	6.09 ± 1.89 (6.02, 6.16)	5.34 ± 1.71 (5.2, 5.48)
Sex		
Male	6.15 ± 1.96 (6.05, 6.25)^a^	5.36 ± 1.56 (5.13, 5.58)^a^
Female	6.04 ± 1.82 (5.95, 6.14)^b^	5.33 ± 1.83 (5.16, 5.50)^b^
Age, years		
18–24	5.68 ± 1.80 (5.46, 5.91)^a^	5.46 ± 1.10 (5.10, 5.83)^a^
25–44	5.89 ± 2.16 (5.76, 6.02)^b^	5.41 ± 0.89 (5.11, 5.70)^b^
45–64	6.13 ± 1.85 (6.03, 6.24)	5.30 ± 1.62 (5.07, 5.54)^b^
≥ 65	6.54 ± 1.44 (6.39, 6.70)	5.29 ± 3.28 (5.09, 5.48)^b^
Education		
Secondary or below	5.93 ± 1.27 (5.83, 6.03)^a^	5.21 ± 1.95 (5.01, 5.40)^a^
Tertiary	6.40 ± 2.55 (6.33, 6.48)	5.58 ± 1.24 (5.41, 5.75)^b^
Employment status		
In paid employment	6.11 ± 2.03 (6.01, 6.20)^a^	5.41 ± 1.22 (5.19, 5.64)^a^
Unemployed	5.30 ± 1.99 (4.96, 5.65)	5.18 ± 1.58 (4.32, 6.04)^b^
Retired	6.43 ± 1.49 (6.30, 6.57)	5.29 ± 2.61 (5.10, 5.49)^b^
Housekeeper	6.07 ± 1.68 (5.82, 6.31)^b^	5.19 ± 1.92 (4.85, 5.54)^b^
Full-time students	5.75 ± 1.73 (5.49, 6.01)	5.43 ± 1.04 (5.06, 5.80)^b^
Monthly household income, HKD		
< 10,000	5.70 ± 1.91 (5.46, 5.93)^a^	4.96 ± 2.46 (4.67, 5.24)^a^
10,000–39,999	5.96 ± 1.61 (5.85, 6.06)	5.13 ± 1.56 (4.91, 5.36)^b^
≥ 40,000	6.55 ± 2.08 (6.45, 6.66)	5.63 ± 1.46 (5.35, 5.90)
Housing type		
Rented	5.92 ± 1.82 (5.81, 6.03)^a^	5.20 ± 1.65 (4.97, 5.43)^a^
Owned	6.21 ± 1.93 (6.12, 6.30)	5.45 ± 1.74 (5.28, 5.63)^b^
Number of cohabitants		
0	6.01 ± 2.21 (5.74, 6.28)^a^	5.51 ± 2.41 (4.88, 6.13)^a^
1–3	6.09 ± 1.86 (6.01, 6.17)^b^	5.31 ± 1.66 (5.16, 5.47)^b^
≥ 4	6.15 ± 1.88 (5.96, 6.34)^b^	5.39 ± 1.66 (5.06, 5.73)^b^

ACC-1, the one-item adversity coping capability scale

CD-RISC2, the 2-item abbreviated version of the Connor-Davidson Resilience Scale

*SD* Standard deviation, *CI* Confidence interval. US$1 = HK$7.8

^a^The reference group

All *P* values for the comparisons with the reference group < 0.05 except for ^b^ > 0.05

Weighted by sex, age, and education distribution of the 2019 Hong Kong general population

## Discussion

This study is the first to assess ACC with a simple and direct question, showing that it was higher in people of older ages, with tertiary education, who were retired, or had higher monthly household income but lower in females and those being unemployed. As hypothesized, ACC was positively associated with resilience, personal happiness and family happiness, wellbeing, and communication quality, and negatively associated with anxiety and depression symptoms, and personal loneliness. ACC-1 was proven to be a valid tool to assess ACC in Hong Kong Chinese population.

Older people and those who were retired showed higher ACC-1 scores. As survivors of the Severe acute respiratory syndrome (SARS) pandemic in 2003 in Hong Kong and experienced more adversities than younger people, they thus had more experiences and confidence in coping with adversities. Older people reported a more optimistic outlook, better adaption, and better mental health during the pandemic [39, 40]. They might show less maladaptive intrapersonal and interpersonal emotion regulation strategies, such as paying more attention to positive emotions and less brooding rumination on negative emotions [40]. Inconsistently, older people reported slightly lower CD-RISC2 scores before the pandemic [19]. Resilience focuses on recovery or bouncing back after adversities, which would be a disadvantage for older people.

People with higher socioeconomic status, including higher education and higher monthly household income, showed higher ACC-1 scores. They usually possess more individual and community resources, such as property, social support, employment flexibility, health knowledge, and neighborhood safety, which influence a higher capability to cope with adversities [41]. The disparities in ACC may result in widening health inequalities in the long run if no special attention is given to those with unfavorable socioeconomic circumstances. Unemployed people showed lower ACC-1 scores, which could be one explanations for the link between increased unemployment and elevated suicide rates amid the pandemic [42]. We also highlighted the sex disparity in ACC, showing that females had a lower level of ACC amid the pandemic, which is corroborated by ours [43] and others’ [44, 45] previous findings that females had higher levels of psychological distress and fear of COVID-19.

We found that people with higher ACC showed better mental health and fewer mental problems, which was in consistent with another study amid the pandemic, reporting that people struggling to cope showed greater anxiety and depression [46]. We additionally showed that ACC was also positively associated with family happiness, wellbeing, and communication quality. These associations could be bidirectional. Well-functioning families could buffer against adversities in the context of COVID-19 through close family organization, effective communication, and shared beliefs [2], which could increase individuals’ perceive ACC. Individuals with higher ACC were found to have better mental health, which could help balance work-family conflicts during the lockdown situations and contribute more to promoting and maintaining family wellbeing amid the pandemic [47].

The significant associations of ACC with stress-related mental health were also consistent with pre-COVID-19 findings [34, 48]. Interventions on coping strategies were proved to be successful to reduce stress-related mental health problems. Thus, ACC should have intervention potential amid the pandemic. Targeted interventions for those with low ACC could be more effective. For example, psychosocial interventions for stress management were found to be more effective in women having lower optimism or who lacked support [49, 50]. Also, a training on coping effectiveness was proven successful in reducing perceived stress, burnout, and anxiety in HIV-positive men [51], and reducing chronic pain in adults with persistent arthritic pain [52]. These training involves disaggregating global stressors, distinguishing malleable aspects of stressors, tailoring particular coping strategies, and selecting and maintaining support resources [53]. Health education promoting coping may be a good strategy to protect vulnerable people from the psychological burdens of the pandemic.

This study is also the first to show evidence in support of the validity of ACC-1 in the general population amid the pandemic. The medium to large positive correlations of ACC-1 with resilience, personal happiness, and family happiness, wellbeing, and communication quality, as well as its negative correlations with anxiety and depressive symptoms and personal loneliness were similar across sex, age, and education, indicating its satisfactory construct validity. ACC-1 had stronger correlations with anxiety symptoms, personal happiness, and family happiness, wellbeing, and communication quality than CD-RISC2, suggesting ACC-1 was different from the measure of resilience. People with favorable socioeconomic status had higher ACC-1 scores, indicating satisfactory known-group validity of ACC-1. ACC-1 additionally contributed to the variations of all measures of mental health and family wellbeing after adjusting for covariates and CD-RISC2, suggesting its incremental validity. ACC and resilience are two distinct yet related concepts [54]. ACC-1 showed stronger correlations with most measures of mental health and family wellbeing than CD-RISC2, as well as a stronger correlation with CD-RISC2-a (adaption) than with CD-RISC2-r (recovery). Since the COVID-19 pandemic from late 2019, people have gradually accepted the concepts of ‘Coexisting with the COVID-19 virus’ and ‘the New normal’ and may therefore report lower scores in the CD-RISC2-r item.

With only one item, ACC-1 minimizes operational and respondent burdens, making it an attractive tool for large-scale studies. Our team designed ACC-1 with a response score ranging from 0 to 10 (an 11-point scale), because it should provide higher discriminating power than a 2-point, 3-point, 4-point, and 5-point scale [55]. It is also easier and more straightforward for respondents to rate out of 10. Moreover, influenced by the Zhongyong thinking from Confucian thought, Chinese people tend to avoid extremities in thinking and acting [56], and are generally less likely to give extreme scores such as no capability or very high capability. Therefore, a wider score range allows for a more precise answer choice than a narrow range. Such response ranges should be further tested in different populations.

This study had some limitations. First, due to the cross-sectional design, causal relationships could not be inferred in this study. However, we identified vulnerable groups that had lower ACC who may need special attention and assistance. Also, test–retest reliability for single-item measures was not included in this study. Second, the study sample consisted of urban Hong Kong Chinese amid the pandemic. It is uncertain whether our results could be generalized to other settings. Third, given the single-item nature of ACC-1, detailed items on coping subscales were not included in the scale, and item response theory analysis could not be applied to refine items or assess internal consistency. Additionally, further research is warranted to verify the validity and reliability of ACC-1 in different cultural backgrounds and contexts. Whether ACC-1 can be used and be sensitive to changes in intervention programs needs further investigation, but a scale of 0 to 10 appears better than scales with more narrow ranges.

## Conclusions

We have first shown that adversity coping capability was associated with better mental health and family wellbeing, and the results support our one-item ACC-1 as a simple and valid measure of ACC. Females and unemployed people showed lower ACC and may need more assistance amid the pandemic. Further research is warranted to verify the validity and reliability of ACC-1 in different cultural backgrounds and contexts.

## Data Availability

The datasets analyzed for the current study are available on request from the corresponding authors. The survey data are not publicly available because our analyses and paper writing on the results are in progress.
